# Uterine Hemorrhage

**Published:** 1861-10

**Authors:** 


### Uterine Hemorrhage.

There is, perhaps, no subject comprised in the catalogue of female maladies which demands the judicious and prompt attention of the physician more than that of Uterine Hemorrhage, and more especially so, when it is connected with the parturient period. And believing that the following summary will greatly aid the efforts of even the experienced practitioners, but more particularly the juveniles of the profession, we submit it to our readers as well worthy their careful perusal.

*Hemorrhage During Labor.*—When the patient is sinking from loss of blood, administer ether-vapor as a *stimulant;* and after the labor, even if the uterus has contracted well, give a dose of secale cornutum. *Part* xiv., *p.* 251.

*Treatment of Post-Partum Hemorrhage.*—Dr. Torbock proposes to arrest flooding by the injection of stimulants into the uterine cavity. He gives two cases in support of the proposed practice.

*Use of Ice in Promoting Uterine Contraction.*—Dr. Mackall, in a communication to the committee of the American Medical Association, states that for several years past he has been in the habit of employing pounded ice in cases of suspended or protracted labor. That when this had been swallowed freely, the pains had immediately returned, the uterus had contracted strongly, and the labor been speedily completed.

In cases where labor pains had been suspended for twelve or twenty-four hours, they have been renewed promptly and efficiently. In cases of inevitable abortion, where the uterine contractions are feeble and inefficient, and where hemorrhage is considerable; in retention of the placenta from imperfect contraction of the uterus, and in cases of alarming hemorrhage after delivery and expulsion of the after-birth, it is equally applicable—in short, whenever the firm contraction of the uterus is desirable, that object will most certainly be attained by the administration of ice.

*Hemorrhage—Post Partum.*—In extreme cases, pass one hand into the uterus; introduce the pipe of an enema apparatus by the side of the arm a little way into the vagina, and inject suddenly ice-cold water, which will pass up into the uterus. By the ordinary plan of injecting the vagina, we are not sure that the injection enters the uterus; but by having one hand introduced into this organ, we learn whether it is really injected, and we ascertain also the effects of the injection. This method is very much better than that of introducing a bladder into the uterus and then distending the former, as it is the influence of the cold, and not the pressure, that we want to employ. *Part* xx., *p.* 194.

*Galvanism in Obstetric Practice.*—Do not employ the apparatus in which two currents are produced, but simply the single current machine; for a direct current tends to produce contraction—an inverse current, paralysis. In using the single current machine, place the positive conductor over the lumbo-sacral region, and carry the other over the abdominal surface, with a gentle friction. In this way powerful uterine contractions may be easily excited.

*Part* xxv., *p.* 278.

*Treatment of Unavoidable Hemorrhage.*—In treating *unavoidable hemorrhage*, attended with exhaustion, Dr. Murphy very much prefers having resource to Dr. Simpson's plan of artificial separation of the placenta, to turning; and thinks this last operation is quite unjustifiable, on Smellie's plea that the woman must not die undelivered—the very attempt to prevent her doing so being, in fact, sometimes the cause of her death. His rules are thus summed up:

1. In cases where no exhaustion has taken place, or where this is only commencing, the child should be turned and delivered the moment the os is sufficiently dilated or dilatable. When this is not so, the placenta should be compressed by the plug, and by the discharge of the liquor amnii, and other means employed to prevent exhaustion, until delivery is practicable.

2. In extreme exhaustion, turning should not be performed, but the placenta should be separated, and the child left undisturbed until decided reaction takes place.

3. When the os is rigid, means should be employed to compress the placenta and increase the action of the uterus, so as to give time for dilatation and turning. Should exhausting hemorrhage, however, come on in the meantime, the placenta should be removed, rather than the hand be forced into the uterus. *Part* xxvi., *p.* 312.

*Hemorrhage after Delivery Accompanied by Severe After-Pains.*—In several cases which Dr. Ramsbotham has met with, there have been most severe after-pains, with great hemorrhage, though the uterus was still firm and comparatively small. His explanation is, that a clot having filled the cavity of the uterus, the womb is unable to expel the offending mass. The more fluid parts are squeezed out, and the firmer remain behind, clinging with such tenacity to the uterine walls as to be almost as difficult of removal as a piece of adherent placenta. The treatment of such cases is to introduce the hand, remove the clot from the cavity of the uterus, and immediately the after-pains and the hemorrhage dependent thereon will cease.

Dr. R. gives the following, among other cases:

My assistance was sought by a medical practitioner, for Mrs. C. H., who had been delivered of her first child three hours. The placenta had been retained by irregular contraction, and removed from the uterus by the hand; previously to which she had lost about two pounds of blood. When I arrived, she was faint, cold, and pale; the pulse was very small; the uterus was excessively hard, and rather larger than natural; there was an uninterrupted draining going on, and she was suffering excruciating pains at intervals, evidently proceeding from uterine contractions. She had taken three drachms of laudanum without relief. I passed my hand into the cavity with considerable ease, notwithstanding the strength of the uterine contractions, and removed a quantity of tough, fibrinous coagula, considerably larger than an orange. She felt immediate freedom from pain, and the discharge ceased almost as suddenly. She became a little faint, but soon rallied; at four she was sleeping comfortably, and her recovery was uninterrupted.

*Part* xxvii., *p.* 180.

*Peculiar Accidental Uterine Hemorrhage*—Sudden syncope or faintness, with great distention and pain of the abdomen, supervening in cases of advanced pregnancy, should lead you to suspect internal uterine hemorrhage.

What would stop the bleeding most effectually for the time till the patient could rally? The coagulum at the mouths of the vessels supported in its position as it would be by the fœtus, membranes, etc. Withdraw this plug, and the hemorrhage would almost certainly return. Therefore, the practice would naturally be to keep the plug there till we saw a fit occasion to allow it to come away. Bringing the child away, and emptying the womb by artificial means, would insure the separation of this plug at a time perhaps when the woman was not fit for such a process. We should say, therefore, in such a case:

1. Give stimulants in moderation, and keep the woman horizontal.

2. If syncope continued, rupture the membranes, give a dose of ergot of rye, and keep up the patient as well as you can by stimulants and food. But don't bring away the child yourself—let the womb expel it, and be ready the moment the child is born to compress the fundus uteri with a cold hand or cold wet towel, so as to compel it to contract rapidly and to expel placenta and coagula. If you save the woman from the first gush of blood, you will probably save her altogether. *Part* xxvii., *p.* 183.

*Galvanism in Obstetric Cases.*—A continual current is by no means so effectual as repeated shocks; these rarely fail to excite muscular contractions, for this stimulus acts directly on muscular fibre. The discs of the apparatus may be covered with thin flannel, and applied on either side of the uterus, the flannel being moistened with water. This plan is equally effectual as the application of the discs to the spine of the cervix uteri.

In cases of hemorrhage before the birth of the child, apply the poles of the galvanic battery to the side of the uterus. *Part* xxix., *p.* 259.

*Galvanism as an Obstetric Agent.*—[Dr. Radford was first led to the use of galvanism in midwifery from observing its value in a case of atony of the bladder. He has used it :]

1st. In cases of tedious labor arising from uterine inertia.

2d. In cases of accidental hemorrhage, either before or after the rupture of the membranes, and especially when exhaustion from loss of blood exists.

3d. In cases of "placenta prævia," in which the practice of detaching the placenta is adopted, and the vital powers are greatly depressed.

4th. In cases of internal flooding before or during labor.

5th. In cases of post-partum floodings.

6th. In cases of hour-glass or irregular contraction of the uterus.

7th. To originate, *de novo*, uterine action, or in cases in which it is desired to induce premature labor.

8th. In cases of abortion, when the indications show the necessity, or justify the expulsion of the ovum.

9th. In cases of asphyxia in infants.

Galvanism is especially advantageous as a general stimulant in all those cases in which the vital powers are extremely depressed from loss of blood. Its beneficial effects are to be observed in the change of the countenance, restoring an animated expression; in its influence on the heart and arteries; in changing the character of respiration; and its warming influence on the general surface.

*Part* xxix., *p.* 268.

*Flooding after Delivery.*—Inject cold water into the womb. Use a Weiss's syringe, and introduce the nozzle of the tube an inch or two fairly into the os uteri.

*Part* xxxi., *p.* 208.

*Hemorrhage after Delivery.*—Generally the more powerfully the uterus contracts after delivery, the less the danger of flooding; not so, however, in all cases; if a portion of the placenta, a fold of the fœtal membranes, or a quantity of coagulated blood, be retained in its cavity, it may be adherent to the internal surface, so that no efforts of the uterus can dislodge it; the more fluid parts may be squeezed away, but the tougher particles remain, and bleeding still go on so as to endanger the life of the patient. By the introduction of the hand, and the withdrawal of the coagula, the agonizing pains and the further loss of blood are at once put a stop to, and instantaneous ease and immediate safety secured. *Part* xxxii., *p.* 221.

*Uterine Hemorrhage.*—When threatened abortion is from a debilitated condition of the uterus, which, in its relaxed condition opens the os, small doses of ergot act as a tonic and close the bleeding orifices of the vessels. If the patient be near the full time, and delivery cannot be prevented, whether a case of placenta prævia, or not, rupture the membranes at one side, and give a good dose of ergot, so as to force the presenting part of the child firmly down upon the bleeding surface, and thus to dam up the stream; if this be not successful, deliver, if you can pass the hand; but if you cannot without much force, plug, until such time as you can deliver.

In women peculiarly liable to flooding, as soon as the child is born, be careful not to interfere unnecessarily during delivery. *Imitate nature*, or rather *let nature alone.* When the head is expelled, don't seize upon it and pull for your life, like a madman, but wait patiently, not only until nature has expelled the shoulders, but the hips also, for the body and legs of the child will stimulate the uterus to action as effectually as the hand of the accoucheur, and thus prevent flooding. But some may say, supposing the head is expelled, and the pains cease, would you not employ traction then? No! certainly not! you must not lend so much as the strength of a finger toward completing the delivery, but "*compel the uterus to do its own work.*" Besides being careful not to interfere in these cases, as soon as the head presses upon the perineum, give a full dose of ergot as a precaution against flooding, and repeat if necessary.

In cases of abortion, if the flow of blood be great, give large and repeated doses of ergot, so that the presenting part may be forced down, to act as an internal plug. In addition to this, it may be advisable to employ the plug, by pushing a piece of alum into the vagina before the plug.

After the child is delivered, the first aim is to secure uterine contractions; it is worse than useless to deliver the placenta if this is not effected; it must be excited by ergot, firmly grasping the uterus, abdominal frictions, ice, the cold and hot douche alternately; if these means are not sufficient, introduce the hand and remove the placenta.

In cases of abortion where the placenta is retained, the general treatment is to trust to time; if there is hemorrhage, plug and give ergot; but there is a great deal of uncertainty about this. The placenta should be removed if possible; some recommend hooking it down with one finger, but this cannot always be done; in such cases, perhaps, a pair of polypus forceps would be best to seize the placenta, twist it round and round again so as to detach it and bring it away.

After the placenta is expelled, there may be hemorrhage from irregular contraction, loss of power and nervous energy, or from some mechanical impediment to contraction, as a clot of blood. In all these cases you must introduce the hand, and when it is contracted, keep it so by external pressure and ergot.

In cases of severe flooding, don't apply the bandage; it is in the way, and prevents more certain manipulations; after all danger has passed, it may be applied for support to the abdomen. *Part* xxxii., *p.* 222.

*Post-Partum Hemorrhage.*—In cases weere women have suffered from flooding in several previous labors, you should give one or two doses of ergot during the last pains which expel the child. Immediately after delivery, in suspicious cases, the hand should be placed on the abdomen, and pressure made until efficient contraction is felt to take place. The uterus should be grasped and held firmly by the hands. The application of cold to the vulva and abdomen, by douching with a wet towel, or dashing the water on, is very effectual in rousing the uterus to contraction. If these means fail, the hand should be washed and introduced into the uterus. In addition to this, if the uterus appear paralyzed, cold water may be injected in a full stream into its cavity, or galvanism may be tried if there be time and convenience. Cold and heat applied alternately are much more efficacious than cold applied alone to excite reflex action. When one reflex surface is exhausted you may appeal to another, dashing cold water on the face, swallowing a gulp of cold water, an enema to the rectum, etc. Whenever coagula collect in the uterus or vigina, they produce irritation, keep up the hemorrhage, and must be turned out with the hand. If the placenta be only partly separated, or remain in the uterus, it must be immediately removed; if the patient is very much reduced, do not wait; pour some brandy down her throat, and proceed at once. *Part* xxxiv., *p.* 233.

*Galvanism in Obstetric Practice.*—Individual shocks applied to the uterus produce no appreciable effects upon it; and a current directed transversely through the organ produces only a partial contraction of it in the direction of the current; but a sustained current of electricity directed longitudinally through the uterus, from the upper portion of the spinal cord, excites the action of the uterus, and singularly enough also accelerates the dilitation of the os uteri. A case of placenta prævia is mentioned, in which several alarming hemorrhages had occurred before labor had commenced. A sustained current of electricity applied in the manner stated for six hours, not only prevented further hemorrhage, but so accelerated the dilitation of the os uteri, that the hand was readily introduced and delivery completed with safety to the patient, although the child, from the extensive separation of the placenta, was still-born. In another case, this mode of treatment speedily induced the expulsion of an organized membrane remaining after an early abortion, the ovum having been previously expelled. The patient was much exhausted by repeated floodings.

*Part* xxxvii., *p.* 271.

